# C1q Nephropathy: The Unique Underrecognized Pathological Entity

**DOI:** 10.1155/2015/490413

**Published:** 2015-11-10

**Authors:** Joe Devasahayam, Gowrishankar Erode-Singaravelu, Zeenat Bhat, Tony Oliver, Arul Chandran, Xu Zeng, Paramesh Dakshinesh, Unni Pillai

**Affiliations:** ^1^University of MO, 1 Hospital Drive, Columbia, MO 65201, USA; ^2^South West Acute Hospital, Enniskillen BT74 4RT, UK; ^3^Wayne State University, 42 W. Warren Avenue, Detroit, MI 48202, USA; ^4^Sanford University, 1305 W. 18th Street, Sioux Falls, SD 57105, USA; ^5^Temple University, 1801 N. Broad Street, Philadelphia, PA 19122, USA; ^6^Presence Covenant Medical Center, 1400 W. Park Street, Urbana, IL 61801, USA; ^7^Ball Memorial Hospital, 2401 W. University Avenue, Muncie, IN 47303, USA

## Abstract

C1q nephropathy is a rare glomerular disease with characteristic mesangial C1q deposition noted on immunofluorescence microscopy. It is histologically defined and poorly understood. Light microscopic features are heterogeneous and comprise minimal change disease (MCD), focal segmental glomerulosclerosis (FSGS), and proliferative glomerulonephritis. Clinical presentation is also diverse, and ranges from asymptomatic hematuria or proteinuria to frank nephritic or nephrotic syndrome in both children and adults. Hypertension and renal insufficiency at the time of diagnosis are common findings. Optimal treatment is not clear and is usually guided by the underlying light microscopic lesion. Corticosteroids are the mainstay of treatment, with immunosuppressive agents reserved for steroid resistant cases. The presence of nephrotic syndrome and FSGS appear to predict adverse outcomes as opposed to favorable outcomes in those with MCD. Further research is needed to establish C1q nephropathy as a universally recognized distinct clinical entity. In this paper, we discuss the current understanding of pathogenesis, histopathology, clinical features, therapeutic options, and outcomes of C1q nephropathy.

## 1. Introduction

C1q nephropathy is a rare form of glomerulopathy first described as a distinct clinic-pathological entity by Jennette and Hipp in 1985 [1]. Its definition is histological and comprises (1) characteristic deposition of C1q in the renal mesangium in a dominant or codominant fashion and (2) the absence of clinical or immunological features of systemic lupus erythematosus (SLE). Exclusion criteria also include type 1 membranoproliferative glomerulonephropathy (MPGN). The prevalence of C1q nephropathy in renal biopsies varies from 0.2 to 16% and appears to be higher in children [2, 3]. It usually presents in children and young adults with either simple proteinuria or frank nephrotic syndrome and is associated with a high proportion of steroid resistance [2, 4]. The clinical and microscopic presentations are quite varied, and the diagnosis is based on histopathology. Likewise, outcomes generally depend on clinical and histological factors. Patients presenting with lower level proteinuria, nephritic syndrome, and histologic variant of minimal change disease (MCD) tend to have favorable outcomes, as opposed to those with nephrotic range proteinuria and focal segmental glomerulosclerosis (FSGS) variant having unfavorable outcomes. We review the pathogenesis, histological findings, clinical features, therapeutic options, and outcomes in patients with C1q nephropathy.

## 2. Complement C1q: Key Component in Complement Pathway

Complements are a heterogeneous group of 40 proteins circulating in the blood stream. They get activated by specific molecules like autoantibodies, immune complexes (classical pathway), carbohydrate molecules in the surface of microorganisms (the MB lectin pathway), or a low grade spontaneous activation called alternate pathway [5]. They play an important role in many diseases, especially immune mediated disorders. Complement targeted drug therapies are available for several diseases. C1 is the first member of the complement system. It forms the first component in the classical pathway. Structurally C1 is a pentamer composed of C1q and two C1r and C1s molecules. The C1q is a 410-kilo dalton glycoprotein molecule. It is produced in various types of cells including monocytes, microglial, dendritic, and endothelial cells. Many other cells including antigen presenting cells, monocytes, glial cells, and macrophages are also capable of synthesizing it [6]. The receptor for the C1q protein is also found in similar cells and plays a role in the classical pathway of the complement activation. A full description of the complement activation pathways is beyond the scope of this paper. It is sufficient to say that, during the classical pathway, C1q recognizes and binds to the immune complex (or to the immunoglobulins IgG and IgM as observed by some authors) and activates the other components of C1. C1q is also known to play a role in the regulation of autoimmune disorders, complications of pregnancy like preeclampsia and eclampsia, and certain malignancies including prostate cancer [7]. Also there have been reports of increased risk of SLE in patients who have hereditary deficiency of C1q [8].

## 3. Pathogenesis

The pathogenesis of C1q nephropathy is still not clear. Specialized C1q receptors which help in the binding of immune complexes are found in the mesangial cells of the kidneys [9]. The detection of C1q complement and immunoglobulin deposition in the glomeruli suggests the possibility of an immune complex mechanism underlying the disease process. However, the exact mechanism by which immune complexes have selective affinity to the renal mesangial cells is uncertain. At present, no specific antigen has been identified. Alternatively, the affinity of C1q molecule to a variety of polyanionic substances including DNA, RNA, viral proteins, gram negative bacteria, and a variety of immune cells may mean that a direct mechanism may exist, and immunoglobulins may just be bystanders in the process. Though the role of podocyte injury in the pathogenesis remains uncertain, the presence of podocyte foot process effacement raises the possibility of “podocytopathy,” at least in a subset of patients [10]. Certain viruses like Epstein Barr virus [11] and BK virus [12] have also been tentatively identified to be associated with C1q nephropathy. Interestingly, C1q dominant deposition has also been noted in allograft kidneys in those without C1q nephropathy in native kidneys, with no apparent clinical significance [13].

## 4. Histological Findings

The histological patterns of C1q nephropathy are heterogeneous as outlined below.

### 4.1. Light Microscopy

C1q nephropathy could be broadly classified into two subtypes on the basis of light microscopy: (1) MCD/FSGS group and (2) immune complex mediated proliferative glomerulonephritis (GN) group. The latter is an umbrella group for several morphological appearances including focal or diffuse mesangial proliferative GN, membranous GN, and membranoproliferative GN. In addition, the FSGS group has three variants, namely, collapsing, cellular, and “not otherwise specified” variants [3]. The proportion of these two groups varies between different notable series of cases (Table 1).

### 4.2. Immunofluorescence Microscopy

**Figure 1:**
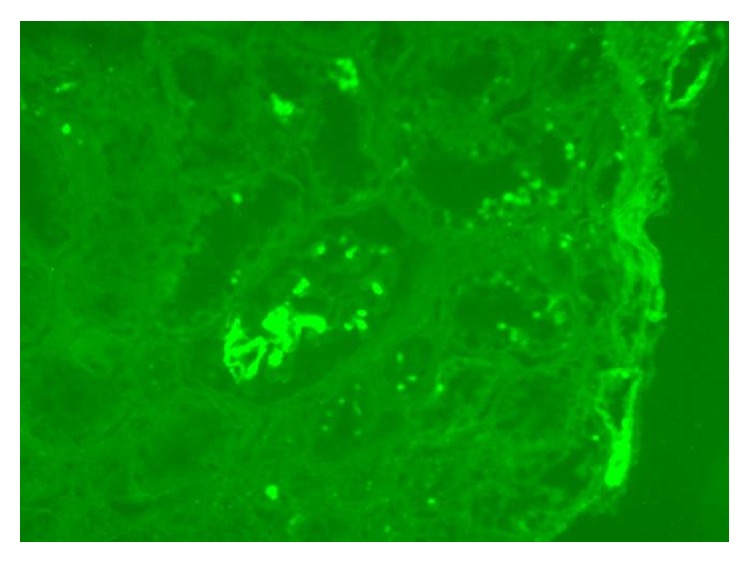
Immunofluorescence study in a patient with C1q nephropathy showing strong mesangial staining.

Immunofluorescence microscopy is more specific than light microscopy. The mainstay of immunofluorescence microscopy is the use of antisera against immunoglobulins or compliment components or even proteins like albumin and fibrinogens. The pattern of staining such as granular, linear, mesangial, or capillary pattern as well as the anatomical location of the staining all would aid in making the specific diagnosis in a renal biopsy. Antiserum against C1q (prepared from goat) is more specific and stains the C1q fragment of the complement component C1. Staining for C1q is evident in all cases of C1q nephropathy, either in dominant or codominant fashion, mainly in the mesangium (Figure 1). Immunoglobulins like IgM and IgG are also usually identified, as they provide ligands for C1q in the immune complex formation. Vizjak et al. [10], in the largest series published so far with 72 cases, reported that the frequencies of IgM, IgG, and IgA were 58%, 48%, and 34%, respectively. In addition, C3 and C4 were also noted at 60% and 25%, respectively. A full house pattern with deposits of IgG, IgM, IgA, C1q, and C3 was found in 30.6% of cases, predominantly in those with proliferative GN morphology. Immunologic staining for C1q may be seen in many glomerular diseases. Jennette and Hipp [16] found high intensity positivity in a high proportion of cases of proliferative lupus nephritis, membranous lupus nephritis, and type 1 membranoproliferative glomerulonephritis (MPGN). These findings formed the basis of their exclusion of SLE and type 1 MPGN in the diagnostic criteria of C1q nephropathy [16].

### 4.3. Electron Microscopy

**Figure 2:**
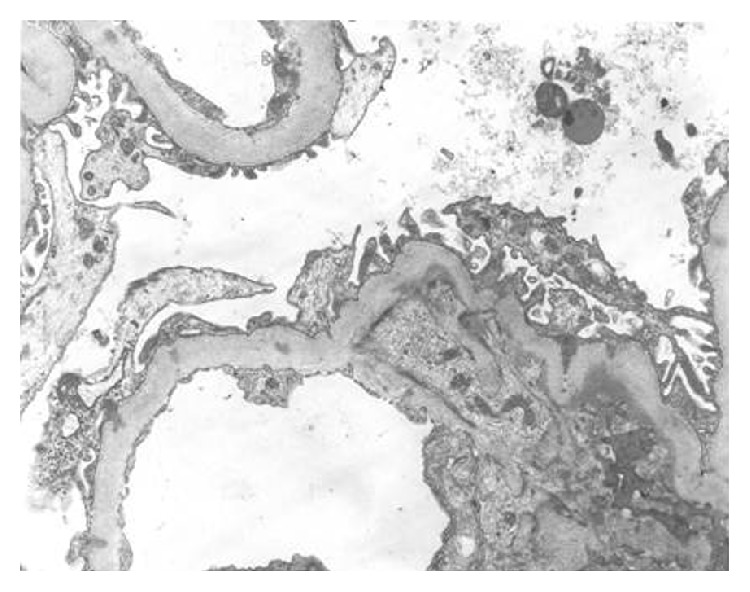
Electron microscopy performed in a patient with C1q nephropathy confirming immunofluorescence findings as mesangial electron dense deposits. In addition, diffuse podocyte foot processes effacement is also identified, indicating podocyte injury.

Diagnostic confirmation of C1q nephropathy is arrived at when amorphous electron dense deposits are demonstrated in the mesangium ± glomerular capillary wall. Podocyte injury can also be noted (Figure 2). These deposits are a consistent finding in all cases irrespective of their light microscopic subtype. Podocyte foot process effacement and cytoskeleton condensation are expressed more commonly in the immune complex mediated subtype. They occur more frequently in patients with nephrotic syndrome or nephrotic range proteinuria than in those with nonnephrotic proteinuria. Rarely, tubuloreticular cytoplasmic inclusions in glomerular and peritubular capillary endothelial cells may also be found.

## 5. Clinical Features

C1q nephropathy is rare, with prevalence ranging from 0.2 to 2.5% [1–3] in biopsies from children and adults and from 2.1 to 9.2% [10, 17] in pediatric biopsies. The prevalence is higher at 16.5% among renal biopsies in children with nephrotic syndrome and persistent proteinuria. There is a slight male preponderance at 68% [10]. It generally affects older children and young adults. Presentation ranges from asymptomatic proteinuria or hematuria to frank nephritic or nephrotic syndromes. Hypertension (35%) and renal insufficiency at the time of diagnosis (5–46%) are common findings [10]. C1q nephropathy presenting as rapidly progressive crescentic glomerulonephritis progressing to end stage renal disease (ESRD) [18] and as acute renal failure requiring renal replacement therapy [19] has also been reported. As discussed above, light microscopy may reveal MCD, FSGS, or immune mediated glomerulonephritis. Interestingly, cases of secondary C1q nephropathy due to viral infection or rheumatoid arthritis have also been reported, with patients exhibiting symptoms of the underlying conditions.

## 6. Treatment

Due to its not-well-understood pathophysiology and varied clinical presentation, C1q nephropathy poses a management challenge. Early specialist consultation is essential. There are no randomized controlled trials that have evaluated the treatment of this condition. Current therapy involves treating the underlying light microscopic lesion, and outcomes vary accordingly. Immunosuppression, commonly in the form of corticosteroids, remains the mainstay of treatment. In steroid resistant cases, pulsed methylprednisolone, cyclophosphamide, azathioprine, Cyclosporine, mycophenolate, and tacrolimus have all been tried separately or in combination therapies with steroids with good response. Rituximab, a monoclonal antibody to CD20, has been used in a couple of patients who failed to respond to steroids with some promising results—one of them achieved normalization of renal function and the other avoided hemodialysis [20].

## 7. Outcomes of C1q Nephropathy in Various Studies

As may be expected, patients with MCD have favorable outcomes compared with those with FSGS. In particular, those presenting with nephrotic syndrome and FSGS often show poor response to corticosteroid treatment [11]. Even in in steroid responders, steroid dependence is a problem even after achieving initial remission. Spontaneous remission is uncommon but has been reported [2, 21]. Despite treatment, some patients will eventually progress to chronic kidney disease and even ESRD requiring lifelong renal replacement therapy.

The following are large, single center series of cases which looked at outcomes in C1q nephropathy.

1. Of 8909 native kidney biopsies processed between 1994 and 2002 at Columbia University in New York, 19 were retrospectively identified to have C1q nephropathy. Sixteen were available for follow-up (mean 27.1 months) of which 12 had received immunosuppressive therapy. Twelve (75%) of them had stable renal function, and 4 (25%) had progressive renal insufficiency. Seven out of 13 patients with proteinuria had partial or complete remission with or without immunosuppressive therapy. Two patients, both of whom had collapsing variant of FSGS, progressed to ESRD with a median renal survival of 81 months [3].
2. In the largest cohort of patients reported yet, a review of 4048 native kidney biopsies from 1985 to 2005 at the University of Ljubljana, Slovenia, revealed C1q nephropathy in 72 biopsies. Of the 11 patients in FSGS group, every one of them presenting with nephrotic syndrome, one-third progressed to ESRD during a mean of 2.9 years of follow-up. In contrast, 77% of the 27 patients with MCD had complete remission of their nephrotic syndrome. Of the 20 patients with proliferative glomerulonephritis, 75% presented as having chronic kidney disease. The majority had stable renal disease after follow-up irrespective of immunosuppressive therapy [10].
3. In a Japanese study which reviewed 16,860 renal biopsies between 1975 and 2004, 61 biopsies were diagnosed to show C1q nephropathy. Mean duration of follow-up was 7.2 years. Three out of 8 patients with FSGS developed ESRD 8 to 15 years after biopsy. In both MCD and FSGS groups, relapse of nephrotic syndrome was common [15].
4. A 2014 study at Great Ormond Street Hospital for Children in London reviewed all biopsies of patients who presented with proteinuria or nephrotic syndrome between 1991 and 2011 and found 35 cases of C1q nephropathy. Thirty children received steroids, and 53% of them were sensitive to therapy. The study found that children with C1q nephropathy of MCD subtype had similar remission rates at four years compared to MCD disease controls, despite more frequent relapses. The long term renal outcome was not significantly different. Most of those with other histological appearances (FSGS, global glomerulosclerosis, or mesangial proliferation) were resistant to steroid therapy, required second-line drugs, and failed to achieve complete remission [4].
5. In another study, 2221 children aged 3 to 15 years underwent percutaneous biopsy between 1975 and 2002 and 30 of them had biopsy proven C1q nephropathy. Of them, 18 children were asymptomatic and the remaining 12 had nephrotic syndrome (NS). The asymptomatic children had more degree of hematuria and the children with NS had more proteinuria. Both the groups had MCD in light microscopy as the underlying diagnosis in the majority of their patients (73%) and the remaining ones had immune mediated GN or FSGN. All these children with NS were treated with prednisolone with or without Cyclosporine. Only 4 of the asymptomatic children received the steroid therapy and the rest of them received dipyridamole. The degree of proteinuria improved in both groups but degree of hematuria improved more in the asymptomatic group [14].
6. Said et al. retrospectively analyzed 24 patients with C1q nephropathy who had renal allograft with the mean age of 31 years. None of these patents had C1q nephropathy or SLE in their native kidneys. These patients developed C1q nephropathy in their transplanted kidneys on an average of 37 months. The light microscopy showed no lesions in about a third of the patients. Almost half of them had their usual antirejection treatment, 4 of them had pulsed steroids, one required plasmapheresis with ACE inhibitor, and one patient was treated with ACE inhibitor alone. Another patient had tacrolimus toxicity and had the dose lowered. The clinical outcome of the remaining patients was not available. The authors concluded that C1q deposition in the allografts was a mere morphological pattern and would have no clinical significance in most patients [13].

## 8. Conclusion

Although three decades have elapsed since C1q nephropathy was first proposed as a distinct clinical entity, it remains poorly understood and controversial. Some authors suggest that it is part of the spectrum of FSGS/MCD. Many reports have described different clinical presentation, histopathology, response to therapy, and outcomes, suggesting that it may be a combination of disease groups than a single entity. However, all published case series have been from single center reviews and appear to be skewed in their demographic profiles. Moreover, the clinical utility of diagnosing C1q nephropathy is yet to be fully established. While there is reasonable evidence to predict poorer outcomes with the presence of FSGS with C1q nephropathy, there have not been any studies which specifically compare clinical characteristics and outcomes of FSGS patients with and without C1q deposition. Routine addition of C1q staining in renal biopsies is not part of current guidelines due to costs and availability involved. Until multicenter, randomized controlled trials are undertaken specifically for C1q nephropathy and results are known, treatment strategy will remain focused on underlying light microscopic pathology, with standard first-line therapy of corticosteroids and immunosuppressive agents in resistant cases.

## Summary

*Features of C1q Nephropathy.* Diagnostic criteria are as follows:

1. characteristic deposition of C1q in renal mesangium in a dominant or codominant fashion,
2. absence of clinical or immunological features of SLE,
3. type 1 MPGN to be excluded.

Histological subtypes are as follows:

1. MCD/FSGS,
2. proliferative glomerulonephritis.

Common presenting features are as follows:

1. asymptomatic hematuria or subnephrotic proteinuria,
2. nephritic syndrome or nephrotic proteinuria,
3. hypertension,
4. renal insufficiency.

Treatment:

1. corticosteroids as first line,
2. immunosuppressive therapy in steroid nonresponders.

Prognosis is as follows:

1. nephrotic syndrome and FSGS—unfavorable outcome,
2. MCD—better outcome.

## Figures and Tables

**Table 1:** 

Series	Total cases	MCD	FSGS	PGN (immune mediated GN)
Markowitz et al. [3]	19	2 (11%)	17 (89%)	0
Fukuma et al. (children) [14]	30	22 (73%)	2 (7%)	6 (20%)
Hisano et al. [15]	61	46 (75%)	8 (13%)	7 (11%)
Vizjak et al. [10]	72	27 (38%)	11 (16%)	20 (28%)
Gunasekara et al. (children) [4]	35	19 (54%)	9 (26%)	7 (20%)
Said et al. (allografts) [13]	24	8 (33%)	5 (21%)	11 (46%)
